# Systematic review and best evidence summary of patient positioning management in the modified lithotomy position during laparoscopic colorectal surgery

**DOI:** 10.1371/journal.pone.0360728

**Published:** 2026-11-12

**Authors:** Jiye Sang, Guojun Zhao, Yanhong Zhang, Jizhong Li, Mingming Zhao

**Affiliations:** 1 Affiliated Hospital of Chengde Medical University, Operating Room, Chengde, China; 2 Yanhong ZHANG: Nursing Department, Affiliated Hospital of Chengde Medical University, Chengde, China; Fujita Health University: Fujita Ika Daigaku, JAPAN

## Abstract

**Objective:**

To systematically retrieve and summarize the best available evidence on patient positioning management for the modified lithotomy position in laparoscopic colorectal surgery, in order to provide an evidence-based reference for standardizing positioning practices.

**Methods:**

Guided by the 6S evidence model, we searched resources such as UpToDate, BMJ Best Practice, the National Institute for Health and Care Excellence (NICE) website, the Registered Nurses’ Association of Ontario (RNAO) website, and the International Guideline Network for relevant clinical decisions, guidelines, evidence summaries, expert consensuses, systematic reviews, and meta – analyses. The search period for guidelines, evidence summaries, and expert consensuses spanned from database inception to October 7, 2025, while systematic reviews were restricted to those published between January 1, 2020, and October 7, 2025. The retrieved literature was appraised for quality, and evidence was extracted and synthesized.This study was registered with the Fudan University Evidence-Based Nursing Center (registration number: ER20259536).

**Results:**

17 publications were included, from which 18 best – practice evidence points were summarized across five domains: preoperative assessment, positioning procedure, positioning – related complications, postoperative handover, and education/training. This summary offers evidence – based guidance on patient positioning in the modified lithotomy position for laparoscopic colorectal surgery, intended to support clinical practice.

## 1 Introduction

The operating room is a high-risk clinical environment in which perioperative risks persist throughout the entire course of patient care [1,2]. Standardized surgical positioning is essential for ensuring safe and effective surgical procedures and places increased demands on operating room nursing staff. Appropriate positioning must not only provide adequate surgical exposure but also minimize the risk of patient injury associated with improper positioning [3]. Surgical positioning requires multidisciplinary collaboration among surgeons, anesthesiologists, anesthesia nurses, and operating room nurses, with operating room nurses playing a central role in safeguarding patient safety [4].

Supine, prone, lateral, and lithotomy positions are standard patient positions commonly used in the operating room [5]. However, with the rapid advancement of surgical techniques, traditional positioning methods may no longer meet the specific demands of complex procedures, often requiring individualized modifications [6]. Notably, positioning-related adverse events continue to occur even when standardized protocols are strictly followed, and most of these injuries are preventable. Under anesthesia, patients experience reduced pain perception, loss of protective reflexes, and muscle relaxation, which increase their vulnerability to positioning-related complications. In particular, the lithotomy position and its modified variants are associated with a significantly increased risk of intraoperative peripheral nerve injury [7], pressure injuries, lower extremity deep vein thrombosis, and even compartment syndrome. Laparoscopic radical resection for colorectal cancer has become the predominant surgical approach due to its advantages, including enhanced visualization, minimal invasiveness, and faster postoperative recovery [8]. The modified lithotomy position, often combined with the Trendelenburg position (head-down tilt), is widely used for this procedure.

The modified lithotomy position represents an optimization of the traditional lithotomy position, with a conceptual shift from a “surgical exposure–centered” approach to one that prioritizes patient safety and comfort while maintaining adequate operative exposure. In the traditional lithotomy position, the hips and knees are highly flexed with the lower legs dependent, which concentrates pressure on the popliteal fossa. This positioning may lead to common peroneal nerve injury and impaired venous return in the lower extremities, thereby increasing the risk of postoperative nerve paralysis, deep vein thrombosis, and other complications. In contrast, the modified lithotomy position incorporates targeted adjustments. First, the degree of hip flexion is reduced, positioning the thighs closer to horizontal to decrease intra-abdominal pressure. More importantly, the support point of the legs is shifted from high-risk areas, such as the popliteal fossa or fibular head, to the calf—specifically the gastrocnemius muscle belly—thereby avoiding major nerves and blood vessels. Additionally, for laparoscopic colorectal surgery, the modified lithotomy position is typically combined with the Trendelenburg position, with a head-down tilt of approximately 15°–25°. This facilitates gravitational displacement of abdominal organs toward the upper abdomen, thereby improving surgical exposure. Previous studies have reported that the incidence of peripheral nerve injury in such positions ranges from 0.16% to 11% [9,10], and the risk is strongly associated with the duration of positioning; notably, each additional hour of positioning may markedly increase the risk of nerve injury [11].

It is important to note that high-quality evidence exclusively examining the modified lithotomy position in laparoscopic colorectal surgery remains limited. The modified lithotomy position shares fundamental anatomical and physiological characteristics with the conventional lithotomy position—including lower extremity elevation, hip and knee flexion, and potential neurovascular compression at the fibular head and popliteal fossa—as well as general perioperative positioning concerns such as pressure injury, venous thromboembolism, and neuromonitoring. Therefore, while this review focuses primarily on positioning management for the modified lithotomy position, the synthesized evidence base also includes applicable findings from the conventional lithotomy position and general surgical positioning, where the underlying pathophysiological mechanisms are considered transferable. The rationale for and limitations of this evidence extension are explicitly discussed in the Discussion section.

Despite these findings, existing literature—both domestic and international—shows considerable variability in standards, procedures, and preventive strategies for complications associated with the modified lithotomy position. Unified, evidence-based guidelines remain lacking. Therefore, this study systematically retrieved and synthesized relevant evidence based on the “6S” evidence-based model, with a primary focus on the modified lithotomy position and inclusion of applicable evidence from related surgical positions where direct evidence was scarce. The aim is to summarize best practices for patient positioning management in the modified lithotomy position during laparoscopic colorectal surgery, with explicit consideration of the applicability of related positioning evidence, thereby providing evidence-based support for clinical nursing practice.

## 2 Materials and methods

### 2.1 Problem identification

The PIPOST framework developed by the Joanna Briggs Institute (JBI) was used to formulate the evidence-based question [12]. P (Population): adult patients (≥18 years) undergoing laparoscopic colorectal cancer surgery; I (Intervention): positioning management interventions for the modified lithotomy position, including multidisciplinary collaboration, risk assessment, equipment selection, positioning techniques, and complication monitoring and prevention. Given the limited direct evidence specific to the modified lithotomy position, evidence from the conventional lithotomy position and general perioperative positioning was also considered where the anatomical, physiological, or pathophysiological mechanisms were deemed applicable (e.g., neurovascular compression, pressure distribution, hemodynamic effects of leg elevation, and thrombosis prevention); P (Professionals): anesthesiologists, surgeons, operating room nurses, and ward nurses; O (Outcomes): (1)patient-level outcomes, including the incidence of positioning-related complications (e.g., peripheral nerve injury, intraoperative acquired pressure injury, lower extremity deep vein thrombosis, compartment syndrome, and postoperative vision loss);(2)healthcare professional–level outcomes, including knowledge awareness and compliance with positioning protocols; and (3) system-level outcomes, including standardization of positioning equipment use, completeness of skin assessment documentation at equipment contact points, and compliance with handover procedures; S (Setting): operating rooms in tertiary hospitals; T (Type of evidence): clinical guidelines, clinical decision support documents, best practice recommendations, evidence summaries, systematic reviews, expert consensus, and original research. Although the primary focus of this review is the modified lithotomy position in laparoscopic colorectal surgery, the evidence base was extended to include relevant studies on the conventional lithotomy position and general surgical positioning. This decision was driven by the scarcity of direct evidence specifically examining the modified lithotomy position. Included studies from related positioning contexts were judged applicable based on anatomical similarity (e.g., lower extremity elevation, hip/knee flexion), shared physiological mechanisms (e.g., compartment pressure dynamics, nerve compression pathways), or position-agnostic pathophysiology (e.g., pressure injury formation, venous stasis). The applicability of such evidence to the modified lithotomy position is critically appraised and explicitly discussed.

This evidence summary was conducted in accordance with the PRISMA 2020 statement. The protocol was prospectively registered with the Fudan University Evidence-Based Nursing Center (registration number: ER20259536) on December 15, 2025), after the literature screening and data extraction had been completed. The predefined search period spanned from database inception to October 7, 2025 for guidelines, evidence summaries, and expert consensuses, and from January 1, 2020 to October 7, 2025 for systematic reviews.

### 2.2 Literature search

Following the “6S” model, a hierarchical search was conducted in UpToDate, BMJ Best Practice, GIN, CMA Infobase, NGC, NICE, Cochrane, AORN Library, PubMed, Medlive, CBM, CNKI, Wanfang, and VIP. Guidelines, evidence summaries, and expert consensus were searched from database inception to October 7, 2025; systematic reviews from January 1, 2020 to October 7, 2025.

Search terms included: (“laparoscopic colorectal surgery” OR “laparoscopic radical resection for colorectal cancer”) AND (“modified lithotomy position” OR “Trendelenburg lithotomy” OR “split-leg lithotomy”) AND (“patient positioning” OR “pressure injury” OR “peripheral nerve injury” OR “compartment syndrome” OR “deep vein thrombosis” OR “postoperative visual loss”).

Two JBI-trained researchers independently conducted the search and cross-checked results.

### 2.3 Inclusion and exclusion criteria

Inclusion criteria: (1) adults undergoing laparoscopic colorectal cancer surgery; (2) studies addressing modified lithotomy positioning management (assessment, positioning, monitoring, handover, training); (3) guidelines, evidence summaries, expert consensus, systematic reviews, meta-analyses, or high-quality original studies; (4) Chinese or English.Exclusion criteria: (1) unavailable full text; (2) interpretations, commentaries, or duplicates; (3) incomplete data.

### 2.4 Literature quality assessment

UpToDate clinical decisions were directly included as high-quality evidence [13]. Guidelines were appraised using AGREE II [14]; scores ≥60% were considered high quality. Expert consensus and systematic reviews were evaluated using the JBI (2016) tool [15] by four trained reviewers, with disagreements resolved by consensus. All procedures followed the registered protocol.

### 2.5 Evidence extraction and synthesis

Two trained researchers independently extracted key data; discrepancies were resolved by an expert. Evidence synthesis was performed by a multidisciplinary panel. Principles included: (1) merging complementary evidence; (2) prioritizing concise, applicable statements; and (3) resolving inconsistencies by consulting original sources, prioritizing high-quality (AGREE II ≥ 60%, JBI ≥ 80%), recent (2023–2025), and consistent evidence.

### 2.6 Evidence grading

Evidence was graded using the JBI pre-grading system (2014) [16] as Grade A (strong) or B (weak) based on FAME criteria. When inconsistencies occurred, priority was given to high-quality, evidence-based, and recent sources.

## 3 Results

### 3.1 Basic characteristics of included literature

The initial search yielded 716 articles: 15 clinical decisions, 73 evidence summaries, 411 guidelines and expert consensus, 72 systematic reviews, and 145 original studies; 391 remained after deduplication; 373 were excluded after reading titles and abstracts; ultimately 17 were included, comprising: 2 Meta-analyses [3,17], 4 expert consensus [7,18–20], 5 clinical practice guidelines [5,21–24], 5 clinical decisions [25–29], and 1 evidence summary [30], Fig 1 and Table 1.

**Fig 1:**
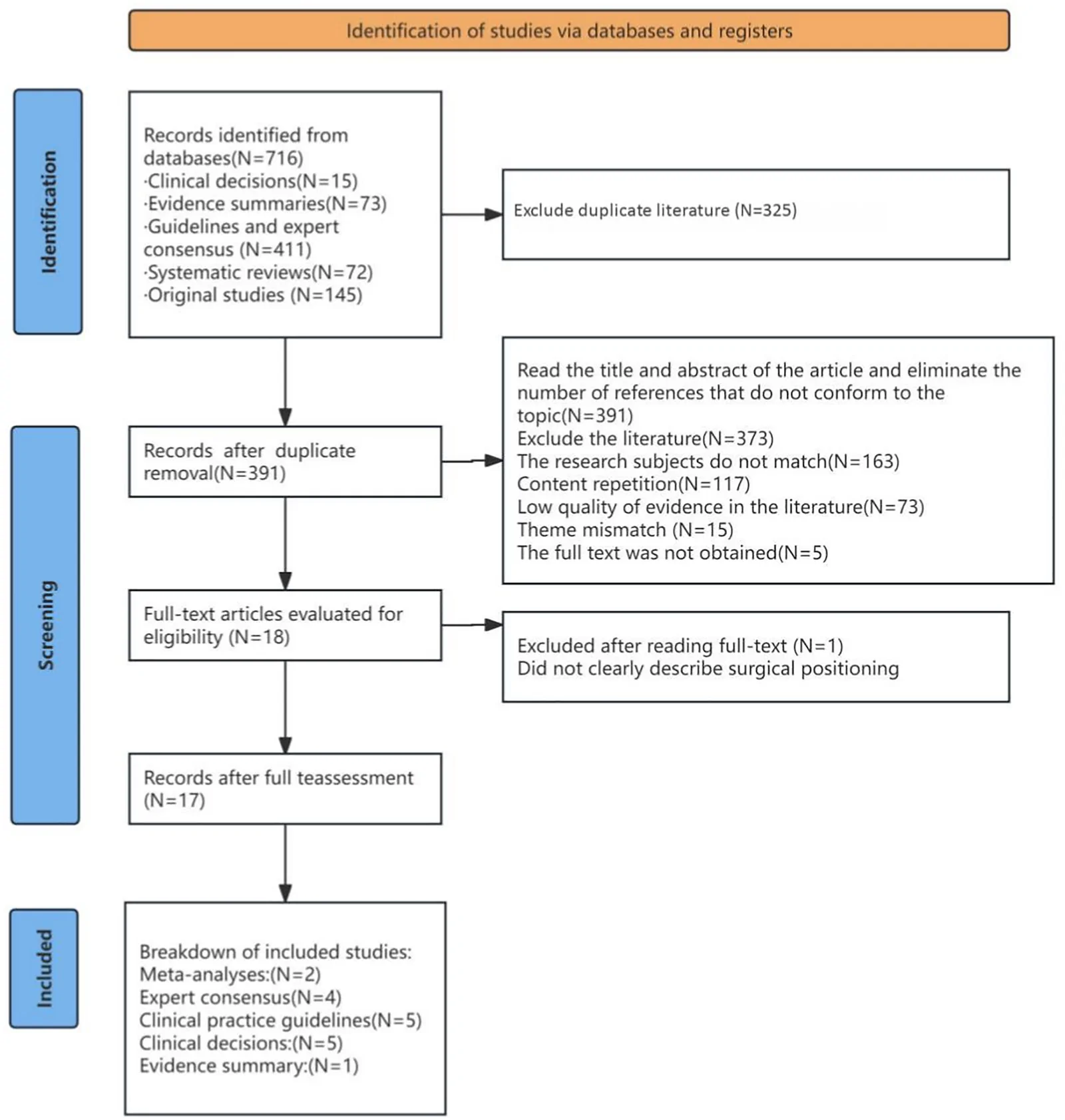
PRISMA flow diagram.

**Table 1:** Basic characteristics of included literature (n = 17).

Included Literature	Publication Year	Source	Type	Topic
Marnie B Welch, et al. [25]	2025	UpToDate	Clinical Decision	Adult Surgical Positioning
Laura B Hemmer, et al. [26]	2025	UpToDate	Clinical Decision	Neuromonitoring in Surgery and Anesthesia
E Mark Hammerberg, et al. [27]	2025	UpToDate	Clinical Decision	Compartment Syndrome
Kevin C Chung [28]	2023	UpToDate	Clinical Decision	Etiology of Compartment Syndrome
Nancy J Newman [29]	2024	UpToDate	Clinical Decision	Vision Loss After Non-Ocular Surgery Anesthesia
AORN [21]	2024	AORN	Guideline	Venous Thrombosis Prevention
Chinese Nursing Association [5]	2024	Chinese Nursing Association	Guideline	Surgical Positioning Management
EPUAP [22]	2021	EPUAP	Guideline	Intraoperative Pressure Injury
AORN [23]	2021	AORN	Guideline	Surgical Positioning
AORN [24]	2023	AORN	Practice Guideline	Patient Surgical Positioning
Ge Weihong, et al. [19]	2025	Wanfang Database	Expert Consensus	Perioperative Venous Thromboembolism Prevention and Treatment
Beijing Nursing Association Operating Room Professional Committee [7]	2025	Wanfang Database	Expert Consensus	Prevention of Positioning-Related Peripheral Nerve Injury in Surgery
Shi Rui, et al. [20]	2024	CNKI	Expert Consensus	Whole-Process Management of Intraoperative Acquired Pressure Injury in Operating Room
China Health Promotion Foundation Thrombosis and Vascular Special Fund Expert Committee [18]	2020	Chinese Medical Journals Full-Text Database	Expert Consensus	Mechanical Prevention of Venous Thromboembolism
Li Bei [30]	2021	CNKI	Evidence Summary	Prevention of Equipment-Related Pressure Injury in Operating Room
Signe Berit Bentsen, et al. [3]	2024	PubMed	Systematic Review	Surgical Positioning and Safety
Cui Luping, et al. [17]	2020	CNKI	Systematic Review	Effect of Intermittent Pneumatic Compression Device Use During Surgery on Postoperative Venous Thrombosis

Note: EPUAP = European Pressure Ulcer Advisory Panel; AORN = Association of PeriOperative Registered Nurses.

### 3.2 Quality assessment results of included literature

#### 3.2.1 Quality assessment results of guidelines (Table 2).

##### 3.2.2 Quality assessment results of evidence summary.

One evidence summary [30] was included, with 3 evidence items extracted, originating from 2 systematic reviews [31,32] and 1 expert consensus [33]. The JBI scores for both systematic reviews were “Yes” (100%), indicating overall high quality. For the expert consensus, item 2 (whether the opinion was derived from authoritative experts in the field) was rated as “Unclear,” while all other items were rated as “Yes,” and it was comprehensively judged as high-quality evidence.

**Table 2:** Quality assessment results of included guidelines (n = 5).

Included Literature	Scope and Purpose	Standardized Scores by Domain (%)	Applicability	Independence	Number of Domains ≥60%	Number of Domains ≥30%	Recommendation Grade
	Stakeholder Involvement	Rigor of Development	Clarity				
AORN [21]	97.2	83.3	87.5	100.0	85.4	91.7	6
Chinese Nursing Association [5]	100.0	94.4	91.7	100.0	89.6	91.7	6
EPUAP [22]	100.0	80.6	85.4	95.0	79.2	94.0	6
AORN [23]	95.0	80.0	94.5	95.6	85.2	100.0	6
AORN [24]	85.7	63.9	62.5	85.7	95.8	100.0	6

#### 3.2.3 Quality assessment results of expert consensus (Table 3).

All 4 expert consensus documents achieved 100% on the remaining 5 items, with only item 6 rated as “Unclear,” meeting the JBI high-quality consensus standard (≥80% of items satisfied).

**Table 3:** Quality assessment results of included expert consensus (n = 4).

Item	China Health Promotion Foundation [18]	Ge Weihong, et al. [19]	Beijing Nursing Association Operating Room Professional Committee [7]	Shi Rui, et al. [20]
1. Is the source of the opinion clearly identified?	Yes	Yes	Yes	Yes
2. Is the opinion derived from influential experts in this field?	Yes	Yes	Yes	Yes
3. Is the proposed opinion centered on the interests of the relevant study population?	Yes	Yes	Yes	Yes
4. Are the stated conclusions based on analytical results? Is the expression of opinions logical?	Yes	Yes	Yes	Yes
5. Are existing literature references cited and accurately indexed?	Yes	Yes	Yes	Yes
6. Are there inconsistencies between the proposed opinion and previous literature?	Unclear	Unclear	Unclear	Unclear

#### 3.2.4 Quality assessment results of systematic reviews (Table 4).

##### 3.3 Evidence summary (Table 5)

## 4 Discussion

A methodological consideration underlying this review is the integration of evidence from the conventional lithotomy position and general surgical positioning into a summary focused on the modified lithotomy position.The modified lithotomy position retains the core anatomical and physiological features of the conventional lithotomy position—specifically lower extremity elevation, hip and knee flexion, and gravitational effects on lower extremity hemodynamics and neurovascular structures—while differing in the degree of hip flexion, the relocation of leg support to the calf, and the routine combination with Trendelenburg tilt. Consequently, evidence regarding nerve compression at the fibular head, saphenous nerve injury at the medial knee, compartment syndrome risk, and impaired venous return is largely transferable from the conventional lithotomy position to the modified variant, provided that these differences are taken into account. Furthermore, evidence concerning pressure injury prevention, neuromonitoring, and venous thromboembolism prophylaxis reflects pathophysiological mechanisms that are largely independent of specific position variations and thus applies similarly across surgical positions, including the modified lithotomy position.

**Table 4:** Quality assessment results of systematic reviews (n = 2).

Item	Signe Berit Bentsen, et al. [3]	Cui Luping, et al. [17]
Item 1: Is the evidence-based question clearly defined?	Yes	Yes
Item 2: Are the inclusion criteria appropriate?	Yes	Yes
Item 3: Is the search strategy appropriate?	Yes	Yes
Item 4: Are the sources of research papers appropriate?	Yes	Yes
Item 5: Are the criteria for literature quality assessment appropriate?	Yes	Yes
Item 6: Was literature quality evaluation conducted independently by 2 or more reviewers?	Yes	Yes
Item 7: Were measures taken to reduce errors during data extraction?	Yes	Partially met
Item 8: Are the methods for synthesizing/merging studies appropriate?	Yes	Yes
Item 9: Was possible publication bias assessed?	Yes	Yes
Item 10: Were recommendations made for policies and/or practice based on reported data?	Yes	Yes
Item 11: Were appropriate suggestions made for specific directions for future research?	Yes	Yes

**Table 5:** Summary of best evidence for patient positioning management in modified lithotomy position during laparoscopic colorectal surgery.

Category	Evidence Content	Evidence Level
Preoperative Assessment and Preparation	1. Preoperative assessment of patient history is required, including high-risk factors such as obesity, age, sex, BMI, lower extremity neurological symptoms, diabetes, peripheral vascular disease, and arthritis [5,25,26]. Special attention should be paid to cardiovascular function to prevent cardiovascular and cerebrovascular accidents caused by the head-down position; comprehensive skin examination and assessment should be conducted combining estimated surgical duration, surgical approach, and preoperative lying position, with focus on bony prominences and areas in contact with equipment [24,25].	5b
	2. Select positioning frames and pads based on patient body type, and evaluate the purpose and effect of prophylactic dressing use [30].	2d
	3. Regularly review and select existing medical equipment in the operating room, assess the purpose and effect of medical equipment use [30], considering factors such as shape, size, patient’s condition, body type, treatment needs, and economic status to select appropriate types, materials, and models of medical equipment [23].	1a
Key Points of Positioning Placement	4. Timing of placement [5,23-25]: Determine the surgical position before sedation or anesthesia induction. Actively pre-adapt to the selected position and assist the patient in adjusting to the most comfortable position. Before skin preparation and draping, usually pre-test the maximum tilt angle required for surgery, especially for severely obese patients, to ensure the patient does not slide, ventilation is smooth, and oxygenation is good [25].	5b
	5. Placement of patients in modified lithotomy position should be completed collaboratively by surgeons, anesthesiologists, and operating room nurses [3,5,23-25].	5b
	6. When placing the modified lithotomy position, hip flexion angle should be < 90°, directing lower extremity weight toward the patient’s soles. Two medical staff should simultaneously place the patient’s legs on leg holders or foot supports. When positioning the hip joint, movements should be slow; when lifting the patient’s legs, first flex the knee to lift the leg to 90° (lower leg and thigh at 90°, thigh and trunk at 90°), then abduct the legs to avoid lumbar spine rotation and hip hyperextension [5,23,25]. In Trendelenburg position, shoulder supports should be placed at the acromioclavicular joint [5]. When using restraint straps, appropriate tightness should be maintained, allowing approximately one finger width [5,24].	5b
	7. Relevant surgical personnel must not lean on or place heavy objects on the patient to avoid increased local pressure [5,22,23].	1b
	8. Intraoperative re-assessment and dynamic adjustment: If lithotomy position placement exceeds 4 hours, the patient should be repositioned in supine position for 20–30 minutes before returning to lithotomy position [23,25].	2a
	9. Once draping is completed, further movement of patient limbs should be avoided to prevent contamination. If intraoperative adjustment of leg holders is needed, operators should be cautious and ensure safety after limb repositioning [5,23].	5b
	10. Removal of legs from lithotomy position also requires multi-person collaboration. After surgery, do not lower both legs simultaneously and rapidly. First help the patient with passive lower extremity movement, then slowly lower legs one by one with approximately 3–5 minutes interval between sides, allowing sufficient time for hemodynamic adaptation and avoiding sudden blood pressure drop [5,7,25].	4a
Positioning Complications and Prevention Strategies	11. Peripheral nerve injury: Common complication, easily affecting ulnar nerve, radial nerve, median nerve, femoral nerve, saphenous nerve, sciatic nerve, common peroneal nerve, lateral femoral cutaneous nerve, and brachial plexus [5,7,26].Prevention measures: ① Upper extremity abduction angle should be ≤ 90°, with soft padding protection at bony prominences such as elbow and wrist [5,7]. ② Select leg holders with soft padding, ensuring no pressure on popliteal fossa and medial knee, lower extremity abduction angle ≤90° [7]. Adjust leg holder position and angle to prevent compression of medial thigh and popliteal fossa, avoiding injury to obturator nerve or popliteal nerve [7]. Lithotomy position leg holder height should equal thigh length, with lower leg and thigh placement angle at 90°. ③ Intraoperative somatosensory evoked potential (SSEP) monitoring may be considered for early detection of nerve ischemia injury [26].	4c
	12. Intraoperatively acquired pressure injury (IAPI) and device-related pressure injury (DRPI).Prevention measures: ① Use positioning pads, place soft padding at bony prominences; when using foot supports, avoid contact between lateral lower leg and any objects or pad with soft material [5,20,23]. When using shoulder supports, avoid carotid artery compression [5,20]. Head-down position easily causes intraoperative downward sliding, increasing friction and shear forces on back skin during sliding [25,34]. Use pressure-reducing dressings at corresponding pressure areas when necessary [20,24]. ② Device-related pressure injury: Check skin at and around medical equipment contact sites preoperatively, intraoperatively, and postoperatively [20,30]. Especially when using IPC, blood pressure cuffs, restraint straps, and pulse oximetry probes, maintain appropriate tightness and observe skin color, temperature, and arterial pulse changes during prolonged use [18,21]. Plastic connections of intravenous catheters, three-way stopcocks, monitor cables and connections are potential sites for pressure injury and should be covered with soft padding [25].	5b
	13. VTE: Surgical patients require prolonged immobilization; surgical trauma, prolonged immobilization, pneumoperitoneum, and lithotomy position are all high-risk factors [17–19,21].Prevention measures: For moderate-to-high risk patients, if no contraindications, routine intraoperative mechanical prevention using intermittent pneumatic compression (IPC) should be implemented [17,19,21]. Postoperative combination with anti-embolism stockings (AES) may be used [19,21] until the patient returns to normal activity [17,18].	5b
	14. Other complications: ① Compartment syndrome: Lithotomy position and head-down position reduce lower extremity perfusion pressure. If relevant signs are detected intraoperatively, repositioning is the primary measure [3,27,28]. ② Exacerbated low back pain: For patients with lumbar disc herniation, preoperative rehearsal of position in awake state helps adjustment, but pain risk remains [25]. ③ Eye injury: Ensure eyelids are closed and covered with dressing intraoperatively to avoid globe compression [29].	5b
Handover and Documentation	15. Preoperatively and postoperatively, operating room, anesthesia recovery room, and ward medical staff are recommended to conduct simple assessment of patient limb neurological function, skin abnormalities, and joint mobility as early as possible and document in records [25].	5b
Education and Training	16. Provide education and training for all medical staff; training content includes: definitions of related complications, epidemiological characteristics, assessment, prevention strategies, and modified lithotomy placement procedures; training methods include brochures, slides with lecture notes, and consultation and Q&A services [5,24,25].	4a
	17. Provide contextualized nursing training, including clinical practice demonstrations and exercises for different patient body types and positions, guiding nursing personnel in the operation and use of related equipment, positioning frames, and operating tables [5,20].	3b
	18. Establish multidisciplinary teams including anesthesiologists, anesthetic nurses, surgeons, and operating room nurses; regularly monitor implementation of prevention measures. Implement assessments for clinical nurses on related content; nursing managers should supervise training and assessment results monthly [20].	2c

This study systematically retrieved and appraised evidence to identify best practices for patient positioning management in the modified lithotomy position during laparoscopic colorectal surgery, resulting in 18 recommendations. These recommendations encompass preoperative risk assessment, standardized positioning, complication prevention, postoperative handover, and multidisciplinary training. As a critical supportive position, the quality of modified lithotomy positioning directly influences surgical efficiency, intraoperative safety, and postoperative recovery. This discussion focuses on four key aspects.

### 4.1 Risk assessment for patients in the modified lithotomy position

Comprehensive and dynamic risk assessment is fundamental to safe positioning management. Preoperative evaluation should prioritize high-risk factors, including obesity, advanced age, diabetes, peripheral vascular disease, and pre-existing lower extremity neurological symptoms. For patients with compromised cardiovascular function, particular attention should be paid to the hemodynamic effects of the Trendelenburg position [2,25,26]. In addition, assessment of surgical duration, skin integrity (especially at bony prominences), and tolerance to preoperative positioning is essential for predicting positioning-related complications [24,25].

Risk assessment should also extend to medical equipment. The selection of positioning devices (e.g., leg holders, positioning frames) should consider design, material, padding, and compatibility with patient anatomy to minimize device-related pressure injury and nerve compression [23,30]. Therefore, a dual-focused approach integrating patient and equipment assessment is necessary to support individualized positioning strategies.

### 4.2 Prevention of positioning-related complications

Medical devices are key contributors to positioning-related injuries. Evidence suggests that: (1) positioning supports should provide adequate stability and cushioning to avoid compression of high-risk areas (e.g., popliteal fossa, fibular head), with additional padding to protect the medial knee and prevent saphenous nerve injury [7]; (2) the tightness of devices such as restraint straps, intermittent pneumatic compression cuffs, and blood pressure cuffs should be carefully controlled (allowing one to two finger widths), with regular skin assessments; and (3) minor pressure sources (e.g., infusion connectors, stopcocks, monitor cables) should be padded appropriately [18,21,25]. Clinically, ergonomically designed and validated equipment should be selected based on patient characteristics, surgical requirements, and cost considerations.

Effective prevention also depends on standardized procedures. Positioning should involve multidisciplinary collaboration, be performed before anesthesia induction, and follow standardized techniques (e.g., knee flexion prior to abduction; hip and knee flexion ≤90°). Leaning on the patient during surgery should be avoided. For procedures exceeding four hours, repositioning or pressure-relief strategies should be considered. Postoperative repositioning should be gradual and coordinated to minimize hemodynamic instability [5,7,23,25]. These measures can be incorporated into checklists and electronic records to ensure standardized and traceable management.

### 4.3 Postoperative handover and documentation

Postoperative assessment and handover are essential components of positioning management. Following transfer to the recovery unit or ward, healthcare providers should promptly assess limb function, skin integrity, and joint mobility, with findings documented in standardized records [25]. This process ensures continuity of care, facilitates early detection of complications, and provides baseline data for subsequent monitoring. Additionally, structured documentation supports effective multidisciplinary communication and enhances patient safety. Therefore, integrating positioning-related assessments into routine handover protocols is critical for improving care quality.

### 4.4 Education and training

Effective implementation of evidence-based practices depends on continuous education and training. Training programs should address complication epidemiology, risk assessment, positioning techniques, and the appropriate use of preventive equipment [5,25,30].

Training should emphasize practical competence through simulation, workshops, and clinical demonstrations, particularly for special populations such as obese patients. Multidisciplinary training teams involving surgery, anesthesia, and nursing are recommended to promote collaboration [30]. Regular training, compliance monitoring, and feedback on adverse events can establish a continuous quality improvement cycle. This structured approach facilitates the integration of patient safety principles into routine practice and reduces positioning-related complications.

## 5 Conclusion

This study synthesizes the best available evidence on patient positioning management in the modified lithotomy position during laparoscopic colorectal surgery and proposes a comprehensive framework encompassing risk assessment, standardized implementation, outcome monitoring, and systematic team training. Clinical practitioners and managers should apply this evidence in the context of local conditions, develop standardized protocols and checklists, and facilitate the translation of evidence into practice to enhance surgical safety and nursing quality.

Future research should prioritize generating direct evidence for the modified lithotomy position, including prospective studies on the hemodynamic and compartment pressure effects of combined Trendelenburg-modified lithotomy positioning, as well as evaluating the effectiveness of novel pressure-relieving materials and intelligent positioning monitoring devices in preventing complications, as well as exploring more effective training and implementation strategies.

## 6 Limitations

This study has several limitations that must be acknowledged. The most notable is the paucity of direct evidence specifically examining the modified lithotomy position in laparoscopic colorectal surgery. Consequently, some of our recommendations were necessarily informed by evidence from the conventional lithotomy position or general surgical positioning. While anatomical and physiological similarities provide a reasonable basis for such extrapolation, important distinctions remain—particularly the routine use of Trendelenburg tilt, reduced hip flexion, and calf-based leg support in the modified position—which may limit the applicability of certain findings to hemodynamic management and lower extremity compartment pressures.

Another limitation is the considerable heterogeneity across the included studies with respect to positioning devices, surgical durations, and institutional protocols. This variability inevitably constrains the extent to which our recommendations can be generalized to specific clinical settings.

It should also be noted that this work represents a best-evidence synthesis rather than a de novo systematic review with meta-analysis. The strength of our recommendations is therefore inherently limited by the quality and quantity of the available primary literature, and our conclusions should be interpreted with this caveat in mind. Finally, restricting the search to English-language publications may have introduced language bias.

## Supporting information

S1 File PRISMA 2020 checklist. Completed PRISMA 2020 checklist for this systematic review.
(PDF)
