# Supplementary material for: Systematic review and best evidence summary of patient positioning management in the modified lithotomy position during laparoscopic colorectal surgery
Source: PLoS One. 2026 Nov 12;21(11):e0360728. doi: 10.1371/journal.pone.0360728 (PMC13759119; doi:10.1371/journal.pone.0360728)
Supplement: Supplementary file 2 [file pone.0360728.s002.docx]

Dear Editor and Reviewers,

Thank you for your careful review and constructive comments on our manuscript entitled "Best Evidence Summary for Patient Positioning in Modified Lithotomy Position during Laparoscopic Colorectal Surgery." We have revised the manuscript substantially to address all concerns. All revisions in the manuscript are highlighted in yellow. Below, we provide a pointbypoint response to each comment.

**Reviewer Comment 1**: Please clarify whether this review specifically focuses on the modified lithotomy position or more broadly on the lithotomy position and perioperative surgical positioning. Although the stated inclusion criteria refer to the modified lithotomy position during laparoscopic colorectal surgery, several included sources appear to address general surgical positioning, venous thromboembolism prevention, pressure injury, neuromonitoring, or other perioperative issues rather than the modified lithotomy position specifically. If the review is intended to include broader evidence from the conventional lithotomy position or general surgical positioning, this should be clearly stated and the applicability of such evidence to the modified lithotomy position should be explained.

**Response:** We thank the reviewer for this perceptive and important observation. The reviewer is correct that several included sources address general surgical positioning or the conventional lithotomy position rather than the modified lithotomy position specifically. This reflects a deliberate, transparent methodological choice driven by the scarcity of direct evidence exclusively examining the modified lithotomy position in laparoscopic colorectal surgery, rather than a scope inconsistency.

We have revised the manuscript to clarify this approach comprehensively as follows:

**Introduction:** We have added a new paragraph (penultimate paragraph) explicitly stating that "highquality evidence exclusively examining the modified lithotomy position in laparoscopic colorectal surgery remains limited. The modified lithotomy position shares fundamental anatomical and physiological characteristics with the conventional lithotomy position—including lower extremity elevation, hip and knee flexion, and potential neurovascular compression at the fibular head and popliteal fossa—as well as general perioperative positioning concerns such as pressure injury, venous thromboembolism, and neuromonitoring. Therefore, while this review focuses primarily on positioning management for the modified lithotomy position, the synthesized evidence base also includes applicable findings from the conventional lithotomy position and general surgical positioning, where the underlying pathophysiological mechanisms are considered transferable. The rationale for and limitations of this evidence extension are explicitly discussed in the Discussion section."

**Methods (PIOST framework):** We have expanded the Intervention (I) component of the PIPOST framework to explicitly state: "Given the limited direct evidence specific to the modified lithotomy position in laparoscopic colorectal surgery, the scope was extended to include applicable evidence from the conventional lithotomy position and general surgical positioning, where the underlying anatomical, physiological, or pathophysiological mechanisms were considered transferable to the modified lithotomy context (e.g., neurovascular compression at the fibular head, pressure distribution dynamics, hemodynamic effects of leg elevation, venous stasis, and thrombosis prevention)."

**Discussion:** We have added a new opening section (now Section 4.1: Applicability of Evidence from Related Surgical Positions) that explicitly discusses three key aspects: (a) the anatomical and physiological similarities between the modified and conventional lithotomy positions that support evidence transferability; (b) the critical differences (reduced hip flexion, relocation of leg support to the calf, routine combination with Trendelenburg tilt) that limit transferability for certain outcomes such as hemodynamic management and compartment syndrome risk; and (c) the positionagnostic nature of evidence regarding pressure injury prevention, neuromonitoring, and VTE prophylaxis, which applies universally across surgical positions. We also explicitly acknowledge the uncertainty introduced by evidence extrapolation.

**Limitations:** We have added a new Limitations subsection acknowledging that "direct evidence specifically examining the modified lithotomy position in laparoscopic colorectal surgery is limited; consequently, some recommendations draw upon evidence from the conventional lithotomy position and general surgical positioning. While the anatomical and physiological similarities support the generalizability of these findings, differences—particularly the routine use of Trendelenburg tilt, reduced hip flexion, and calfbased leg support in the modified position—may affect the direct transferability of certain conclusions."

**Conclusion:** We have revised the Conclusion to clarify that the framework "incorporates applicable evidence from the conventional lithotomy position and general surgical positioning where direct evidence was lacking," and we have updated future research directions to prioritize generating direct evidence for the modified lithotomy position.

We believe these revisions transparently address the reviewer's concern, clearly distinguish between direct and indirect evidence, and explain the applicability rationale without artificially imposing a formal evidence hierarchy that was not part of the original methodological design.

**Reviewer Comment 2:** The study selection process should be presented clearly in a PRISMA flow diagram. According to the manuscript, 716 records were initially identified, 391 remained after duplicate removal, 373 were excluded after title and abstract screening, leaving 17 publications ultimately included. The reason for exclusion of the remaining record should be specified. More generally, the reasons for exclusion at each stage, particularly after fulltext assessment, should be clearly reported. The authors should also explain why several general surgical positioning references were included despite the stated inclusion criterion of modified lithotomy positioning during laparoscopic colorectal surgery.

**Response:** We thank the reviewer for these important methodological comments. We have addressed each aspect of this comment as follows:

2.1. PRISMA flow diagram: We have added a PRISMA 2020 flow diagram as Figure 1 to clearly present the study selection process at each stage, following the standard visual format. The diagram depicts the complete flow from identification (N=716) through screening, eligibility assessment, and final inclusion (N=17), including all exclusion points.

2.2. Missing data point and fulltext exclusion clarified: We apologize for the omission in the original manuscript. The reviewer correctly identified that the data chain was incomplete. The correct numbers are: of the 391 records remaining after deduplication, 373 were excluded at the title and abstract stage, leaving 18 records that underwent fulltext assessment. One record was excluded at the fulltext stage because it did not clearly describe surgical positioning, making it impossible to determine its applicability to the modified lithotomy position or related positioning contexts. Consequently, 17 publications were ultimately included. We have revised the Methods section to explicitly report these corrected numbers.

2.3. Table 1 — Fulltext exclusion documented: We have added Table 1 documenting the record excluded at the fulltext assessment stage, including the author, year, and specific reason for exclusion ("Did not clearly describe surgical positioning; the positioning method was inadequately reported to determine applicability to the modified lithotomy position or related surgical positioning contexts"). This addresses the reviewer's request that the reason for exclusion of the remaining record be specified.

2.4. Reasons for exclusion at each stage clearly reported: We have revised the Methods section to clearly report the reasons for exclusion at each stage:

Identification stage: 325 duplicate records were removed.

Screening stage: 373 records were excluded after title and abstract screening due to wrong population (e.g., pediatric or noncolorectal surgery), wrong intervention (e.g., nonlithotomy positioning without transferable mechanisms), or wrong study design (e.g., narrative reviews or case reports not meeting the PIPOST criteria).

Eligibility stage: 1 record was excluded after fulltext assessment because it did not clearly describe surgical positioning.

2.5. Inclusion of general surgical positioning references: Please see our detailed response to Comment 1 above. In brief, we have revised the PIPOST intervention criteria, Introduction, and Discussion to explicitly explain that applicable evidence from the conventional lithotomy position and general surgical positioning was included where anatomical, physiological, or pathophysiological mechanisms were transferable to the modified lithotomy context. This pragmatic extension of the evidence base was necessitated by the limited volume of direct evidence specific to the modified lithotomy position in laparoscopic colorectal surgery. The PRISMA flow diagram also includes a note clarifying the evidence scope of the included studies.

**Reviewer Comment 3:** The registration status of this review should be clarified. The manuscript states that the study was registered with the Fudan University EvidenceBased Nursing Center (registration number: ER20259536), but it is unclear whether this registration was prospective or retrospective. If the registration was completed after the commencement of the review, this should be explicitly disclosed, along with an explanation of the circumstances and confirmation that no substantive methodological amendments were made between the start of screening and the registration date.

**Response:** We thank the reviewer for raising this important issue regarding research transparency. We acknowledge that the registration was indeed retrospective, and we apologize for not having made this explicit in the original manuscript. We have revised the manuscript to provide full transparency regarding the registration status as follows:

**Methods —** Protocol and Registration: We have revised this subsection to state: "The review protocol was registered with the Fudan University EvidenceBased Nursing Center (registration number: ER20259536) on December 15, 2025.

**Abstract:** We have added the following statement to the Abstract: "Registration: This review was registered with the Fudan University EvidenceBased Nursing Center (registration number ER20259536; registration date: December 15, 2025. This registration was retrospective."

We confirm that the review protocol was fully finalized before the commencement of literature screening, and no substantive changes to the methodology (including the research question, PIPOST framework, search strategy, inclusion/exclusion criteria, or quality appraisal methods) were made between the start of screening and the registration date. The retrospective registration does not affect the methodological rigor or the validity of the findings presented in this review. We appreciate the reviewer's emphasis on transparency, and we believe these revisions appropriately address this concern.

**Reviewer Comment 4:** Although the total number of included publications is reported as 17, the reference numbers and composition of the included studies are not consistent among the Results section, Table 1, and Table 2. For example, the guideline references listed in the Results do not fully correspond to those presented in the tables. In addition, the Abstract reports 20 evidence points, whereas Table 5 and the Discussion describe 18 recommendations. The authors should carefully recheck the entire process of study selection, quality assessment, evidence extraction, and evidence synthesis, and ensure consistency among the main text, tables, and reference numbering.

**Response:** We thank the reviewer for this careful observation. We have thoroughly rechecked the entire process of study selection, quality assessment, evidence extraction, and evidence synthesis. All inconsistencies among the Results section, tables, and reference numbering have been carefully corrected and verified. Specifically, the reference numbers and composition of included studies are now fully consistent across the Results, Table 1, and Table 2; and the number of recommendations has been unified to 18 throughout the Abstract, Table 5, and the Discussion. We apologize for these oversights and confirm that the final manuscript is internally consistent.

We hope that these revisions satisfactorily address all of the reviewers' concerns and strengthen the methodological transparency and rigor of our manuscript. We appreciate the opportunity to improve the quality of our work.

Sincerely,

Jiye SANG

1057067874@qq.com
