# Supplementary material for: Systematic review and best evidence summary of patient positioning management in the modified lithotomy position during laparoscopic colorectal surgery
Source: PLoS One. 2026 Nov 12;21(11):e0360728. doi: 10.1371/journal.pone.0360728 (PMC13759119; doi:10.1371/journal.pone.0360728)
Supplement: Supplementary file 3 [file pone.0360728.s003.docx]

Response to Editor and Reviewers

**Manuscript title:** Systematic Review and Best Evidence Summary of Patient Positioning Management in the Modified Lithotomy Position during Laparoscopic Colorectal Surgery

**Corresponding author:**Jiye Sang

**Email:** 1057067874@qq.com

Dear Editor,

We sincerely thank you and the reviewers for your thorough evaluation of our manuscript and for the constructive comments. We have carefully addressed all the points raised and revised the manuscript accordingly. Below is our point-by-point response.

**Response to Editor:**

***Comment:*** *In the response letter, the authors state that the registration was retrospective, whereas the Methods section describes the protocol as prospectively registered prior to literature screening and data extraction. Please confirm the actual registration timing and revise the relevant statements accordingly to ensure consistency between the response letter and the manuscript.*

**Response:** We thank the Editor for identifying this inconsistency. We confirm that the protocol was retrospectively registered with the Fudan University Evidence-Based Nursing Center (registration number: ER20259536) on December 15, 2025, after the literature screening and data extraction had been completed. The previous response letter incorrectly described the registration as prospective. We have revised the Methods section accordingly. The corrected text now reads:

“This evidence summary was conducted in accordance with the PRISMA 2020 statement. The protocol was retrospectively registered with the Fudan University Evidence-Based Nursing Center (registration number: ER20259536) on December 15, 2025, after the literature screening and data extraction had been completed. The predefined search period spanned from database inception to October 7, 2025 for guidelines, evidence summaries, and expert consensuses, and from January 1, 2020 to October 7, 2025 for systematic reviews.”

This ensures consistency between the manuscript and the response letter.

**Response to Reviewer #1:**

We thank Reviewer #1 for the positive evaluation and for the helpful minor corrections.

***Comment 1:*** *References 9–13: Please update the proxy/institutional URLs to standard, official UpToDate URLs or standard citation formats.*

**Response:** All proxy/institutional URLs have been removed and replaced with official UpToDate URLs, formatted in PLOS ONE (Vancouver) style:

9. Welch MB. Patient positioning for surgery and anesthesia in adults [Internet]. UpToDate; 2024 Nov 5 [cited 2026 Sep 14]. Available from: https://www.uptodate.com/contents/patient-positioning-for-surgery-and-anesthesia-in-adults

10. Hemmer LB. Neuromonitoring in surgery and anesthesia [Internet]. UpToDate; [cited 2026 Sep 14]. Available from: https://www.uptodate.com/contents/neuromonitoring-in-surgery-and-anesthesia

11. Hammerberg EM. Acute compartment syndrome of the extremities [Internet]. UpToDate; 2025 Aug 12 [cited 2026 Sep 14]. Available from: https://www.uptodate.com/contents/acute-compartment-syndrome-of-the-extremities

12. Chung KC. Pathophysiology, classification, and causes of acute extremity compartment syndrome [Internet]. UpToDate; 2023 Dec 13 [cited 2026 Sep 14]. Available from: https://www.uptodate.com/contents/pathophysiology-classification-and-causes-of-acute-extremity-compartment-syndrome

13. Newman NJ, Roth S. Postoperative visual loss after anesthesia for nonocular surgery [Internet]. UpToDate; [cited 2026 Sep 14]. Available from: https://www.uptodate.com/contents/postoperative-visual-loss-after-anesthesia-for-nonocular-surgery

***Comment 2:*** *Reference 27: The citation details at the end are incomplete.*

**Response:** Reference 27 has been completed as follows:

27. Shi R, Zhang R, Li HY, He JQ, Chen YC, An JJ, et al.; Guangdong Nursing Association Operating Room Nursing Committee. Expert consensus on whole-process management of intraoperatively acquired pressure injuries in the operating room. Journal of Nursing. 2024;31(19):58–64. DOI: 10.16460/j.issn1008-9969.2024.19.058.

***Comment3:*** *Figure 1 (PRISMA Flowchart): “Identifacation” → “Identification”.*

**Response:** The typo has been corrected to “Identification” in the revised Figure 1.

***Comment 4:*** *Fix the duplicated label “Records after duplicate removal” in the middle flow boxes.*

**Response:** The duplicated label has been removed; Figure 1 now contains a single correct “Records after duplicate removal” box and accurately represents the screening process. The revised figure has been uploaded.

All authors have reviewed and approved the revised manuscript. We confirm there are no conflicts of interest. Thank you again for your time and guidance.

Sincerely,

Sang Jiye
