# Supplementary material for: Systematic review and best evidence summary of patient positioning management in the modified lithotomy position during laparoscopic colorectal surgery
Source: PLoS One. 2026 Nov 12;21(11):e0360728. doi: 10.1371/journal.pone.0360728 (PMC13759119; doi:10.1371/journal.pone.0360728)
Supplement: Peer review history

# Peer review: Decision Letter 0

**DOI:** 10.1371/journal.pone.0360728.r001

**Authors:** Tsutomu Kumamoto

17 Aug 2026

Dear Dr. Jiye SANG,

Thank you for submitting your manuscript to PLOS ONE. After careful consideration, we feel that it has merit but does not fully meet PLOS ONE’s publication criteria as it currently stands. Therefore, we invite you to submit a revised version of the manuscript that addresses the points raised during the review process.

Please submit your revised manuscript by Oct 16 2026 11:59PM. If you will need more time than this to complete your revisions, please reply to this message or contact the journal office at plosone@plos.org. When you're ready to submit your revision, log on to https://www.editorialmanager.com/pone/ and select the 'Submissions Needing Revision' folder to locate your manuscript file.

- A letter that responds to each point raised by the academic editor and reviewer(s). You should upload this letter as a separate file labeled 'Response to Reviewers'.
- A marked-up copy of your manuscript that highlights changes made to the original version. You should upload this as a separate file labeled 'Revised Manuscript with Track Changes'.
- An unmarked version of your revised paper without tracked changes. You should upload this as a separate file labeled 'Manuscript'.

If you would like to make changes to your financial disclosure, please include your updated statement in your cover letter. Guidelines for resubmitting your figure files are available below the reviewer comments at the end of this letter.

If applicable, we recommend that you deposit your laboratory protocols in protocols.io to enhance the reproducibility of your results. Protocols.io assigns your protocol its own identifier (DOI) so that it can be cited independently in the future. For instructions see: https://journals.plos.org/plosone/s/submission-guidelines#loc-laboratory-protocols. Additionally, PLOS ONE offers an option for publishing peer-reviewed Lab Protocol articles, which describe protocols hosted on protocols.io. Read more information on sharing protocols at https://plos.org/protocols?utm_medium=editorial-email&utm_source=authorletters&utm_campaign=protocols.

As the corresponding author, your ORCID iD is verified in the submission system and will appear in the published article. PLOS supports the use of ORCID, and we encourage all coauthors to register for an ORCID iD and use it as well. Please encourage your coauthors to verify their ORCID iD within the submission system before final acceptance, as unverified ORCID iDs will not appear in the published article. *Only*  the individual author can complete the verification step; PLOS staff *cannot*  verify ORCID iDs on behalf of authors.

We look forward to receiving your revised manuscript.

Kind regards,

Tsutomu Kumamoto

Academic Editor

PLOS One

Journal Requirements:

When submitting your revision, we need you to address these additional requirements.

1. Please ensure that your manuscript meets PLOS ONE's style requirements, including those for file naming. The PLOS ONE style templates can be found at

https://journals.plos.org/plosone/s/file?id=wjVg/PLOSOne_formatting_sample_main_body.pdf and

https://journals.plos.org/plosone/s/file?id=ba62/PLOSOne_formatting_sample_title_authors_affiliations.pdf

2. During your revisions, please confirm whether the wording in the title is correct and update it in the manuscript file and online submission information if needed. Specifically, include “systematic review in the title.

3. Please ensure that you have uploaded your PRISMA flowchart as Figure 1, or else explain why this is not possible. Blank flowcharts can be found here: http://www.prisma-statement.org

4. Please include captions for your Supporting Information files at the end of your manuscript, and update any in-text citations to match accordingly. Please see our Supporting Information guidelines for more information: http://journals.plos.org/plosone/s/supporting-information.

5. Thank you for stating the following financial disclosure:

"Medical Science Research Project of Hebei（20242175）

Medical Science Research Project of Hebei（20220418）"

Please state what role the funders took in the study.  If the funders had no role, please state: "The funders had no role in study design, data collection and analysis, decision to publish, or preparation of the manuscript."

If this statement is not correct you must amend it as needed.

Please include this amended Role of Funder statement in your cover letter; we will change the online submission form on your behalf.

6. When completing the data availability statement of the submission form, you indicated that you will make your data available on acceptance. We strongly recommend all authors decide on a data sharing plan before acceptance, as the process can be lengthy and hold up publication timelines. Please note that, though access restrictions are acceptable now, your entire data will need to be made freely accessible if your manuscript is accepted for publication. This policy applies to all data except where public deposition would breach compliance with the protocol approved by your research ethics board. If you are unable to adhere to our open data policy, please kindly revise your statement to explain your reasoning and we will seek the editor's input on an exemption. Please be assured that, once you have provided your new statement, the assessment of your exemption will not hold up the peer review process.

7. If the reviewer comments include a recommendation to cite specific previously published works, please review and evaluate these publications to determine whether they are relevant and should be cited. There is no requirement to cite these works unless the editor has indicated otherwise.

Editor's comments:

The manuscript addresses a clinically relevant topic and has the potential to provide useful guidance for perioperative positioning during laparoscopic colorectal surgery. However, several methodological and reporting issues require clarification before the manuscript can be considered for publication.

Scope of the review

Please clarify whether this review specifically focuses on the modified lithotomy position or more broadly on the lithotomy position and perioperative surgical positioning. Although the stated inclusion criteria refer to the modified lithotomy position during laparoscopic colorectal surgery, several included sources appear to address general surgical positioning, venous thromboembolism prevention, pressure injury, neuromonitoring, or other perioperative issues rather than the modified lithotomy position specifically. If the review is intended to include broader evidence from the conventional lithotomy position or general surgical positioning, this should be clearly stated and the applicability of such evidence to the modified lithotomy position should be explained.

Title and study design

The manuscript represents a systematic evidence review/synthesis rather than a conventional original clinical study. Although systematic reviews may be submitted under the Research Article category in PLOS ONE, the study design should be clearly reflected in the title. The authors should therefore consider revising the title to indicate that this is a systematic review or other appropriate form of evidence synthesis. In addition, the title should accurately reflect whether the evidence is restricted to the modified lithotomy position or includes broader evidence regarding lithotomy or surgical positioning.

Study selection process

The study selection process should be presented clearly in a PRISMA flow diagram. According to the manuscript, 716 records were initially identified, 391 remained after duplicate removal, 373 were excluded after title and abstract screening, leaving 18 records, whereas only 17 publications were ultimately included. The reason for exclusion of the remaining record should be specified. More generally, the reasons for exclusion at each stage, particularly after full-text assessment, should be clearly reported. The authors should also explain why several general surgical positioning references were included despite the stated inclusion criterion of modified lithotomy positioning during laparoscopic colorectal surgery.

Inconsistencies in the included literature and evidence synthesis

Although the total number of included publications is reported as 17, the reference numbers and composition of the included studies are not consistent among the Results section, Table 1, and Table 2. For example, the guideline references listed in the Results do not fully correspond to those presented in the tables. In addition, the Abstract reports 20 evidence points, whereas Table 5 and the Discussion describe 18 recommendations. The authors should carefully recheck the entire process of study selection, quality assessment, evidence extraction, and evidence synthesis, and ensure consistency among the main text, tables, and reference numbering.

Registration and protocol

The authors state that the review was registered with the Fudan University Evidence-Based Nursing Center (registration number ER20259536). Please clarify whether this registration was prospective and provide the date of registration. If the registration record or review protocol is publicly accessible, please provide the relevant information. The authors should also clarify whether the protocol was finalized before literature screening and evidence extraction. In addition, the registration number should be included in the Abstract, and the protocol should be provided as Supporting Information where appropriate.

Reviewer's comments:

Reviewer's Responses to Questions

**Comments to the Author**

1. Is the manuscript technically sound, and do the data support the conclusions?

Reviewer #1: Partly

**********

2. Has the statistical analysis been performed appropriately and rigorously?

Reviewer #1: N/A

**********

3. Have the authors made all data underlying the findings in their manuscript fully available?

The PLOS Data policy

Reviewer #1: Yes

**********

4. Is the manuscript presented in an intelligible fashion and written in standard English?

Reviewer #1: Yes

**********

Reviewer #1: The manuscript addresses an important and clinically relevant topic and provides a useful summary of evidence regarding patient positioning during laparoscopic colorectal surgery. However, some methodological issues should be addressed before publication. The authors should clarify the selection and hierarchy of evidence sources, provide a more consistent explanation of the evidence grading and strength of recommendations, and ensure that specific numerical recommendations are adequately supported by the underlying evidence. In addition, the discrepancy between the reported 20 evidence points and 18 recommendations should be clarified. Overall, the manuscript is potentially valuable but requires further methodological clarification and revision.

**********

what does this mean?). If published, this will include your full peer review and any attached files.

If you choose “no”, your identity will remain anonymous but your review may still be made public.

**Do you want your identity to be public for this peer review?**  For information about this choice, including consent withdrawal, please see our Privacy Policy

Reviewer #1: No

**********

[NOTE: If reviewer comments were submitted as an attachment file, they will be attached to this email and accessible via the submission site. Please log into your account, locate the manuscript record, and check for the action link "View Attachments". If this link does not appear, there are no attachment files.]

To ensure your figures meet our technical requirements, please review our figure guidelines: https://journals.plos.org/plosone/s/figures

You may also use PLOS’s free figure tool, NAAS, to help you prepare publication quality figures: https://journals.plos.org/plosone/s/figures#loc-tools-for-figure-preparation.

NAAS will assess whether your figures meet our technical requirements by comparing each figure against our figure specifications.

# Author comment: Author response to Decision Letter 1

**DOI:** 10.1371/journal.pone.0360728.r002

21 Aug 2026

Dear Editor and Reviewers,

Thank you for your careful review and constructive comments on our manuscript entitled "Best Evidence Summary for Patient Positioning in Modified Lithotomy Position during Laparoscopic Colorectal Surgery." We have revised the manuscript substantially to address all concerns. All revisions in the manuscript are highlighted in yellow. Below, we provide a pointbypoint response to each comment.

Reviewer Comment 1: Please clarify whether this review specifically focuses on the modified lithotomy position or more broadly on the lithotomy position and perioperative surgical positioning. Although the stated inclusion criteria refer to the modified lithotomy position during laparoscopic colorectal surgery, several included sources appear to address general surgical positioning, venous thromboembolism prevention, pressure injury, neuromonitoring, or other perioperative issues rather than the modified lithotomy position specifically. If the review is intended to include broader evidence from the conventional lithotomy position or general surgical positioning, this should be clearly stated and the applicability of such evidence to the modified lithotomy position should be explained.

Response: We thank the reviewer for this perceptive and important observation. The reviewer is correct that several included sources address general surgical positioning or the conventional lithotomy position rather than the modified lithotomy position specifically. This reflects a deliberate, transparent methodological choice driven by the scarcity of direct evidence exclusively examining the modified lithotomy position in laparoscopic colorectal surgery, rather than a scope inconsistency.

We have revised the manuscript to clarify this approach comprehensively as follows:

Introduction: We have added a new paragraph (penultimate paragraph) explicitly stating that "highquality evidence exclusively examining the modified lithotomy position in laparoscopic colorectal surgery remains limited. The modified lithotomy position shares fundamental anatomical and physiological characteristics with the conventional lithotomy position—including lower extremity elevation, hip and knee flexion, and potential neurovascular compression at the fibular head and popliteal fossa—as well as general perioperative positioning concerns such as pressure injury, venous thromboembolism, and neuromonitoring. Therefore, while this review focuses primarily on positioning management for the modified lithotomy position, the synthesized evidence base also includes applicable findings from the conventional lithotomy position and general surgical positioning, where the underlying pathophysiological mechanisms are considered transferable. The rationale for and limitations of this evidence extension are explicitly discussed in the Discussion section."

Methods (PIOST framework): We have expanded the Intervention (I) component of the PIPOST framework to explicitly state: "Given the limited direct evidence specific to the modified lithotomy position in laparoscopic colorectal surgery, the scope was extended to include applicable evidence from the conventional lithotomy position and general surgical positioning, where the underlying anatomical, physiological, or pathophysiological mechanisms were considered transferable to the modified lithotomy context (e.g., neurovascular compression at the fibular head, pressure distribution dynamics, hemodynamic effects of leg elevation, venous stasis, and thrombosis prevention)."

Discussion: We have added a new opening section (now Section 4.1: Applicability of Evidence from Related Surgical Positions) that explicitly discusses three key aspects: (a) the anatomical and physiological similarities between the modified and conventional lithotomy positions that support evidence transferability; (b) the critical differences (reduced hip flexion, relocation of leg support to the calf, routine combination with Trendelenburg tilt) that limit transferability for certain outcomes such as hemodynamic management and compartment syndrome risk; and (c) the positionagnostic nature of evidence regarding pressure injury prevention, neuromonitoring, and VTE prophylaxis, which applies universally across surgical positions. We also explicitly acknowledge the uncertainty introduced by evidence extrapolation.

Limitations: We have added a new Limitations subsection acknowledging that "direct evidence specifically examining the modified lithotomy position in laparoscopic colorectal surgery is limited; consequently, some recommendations draw upon evidence from the conventional lithotomy position and general surgical positioning. While the anatomical and physiological similarities support the generalizability of these findings, differences—particularly the routine use of Trendelenburg tilt, reduced hip flexion, and calfbased leg support in the modified position—may affect the direct transferability of certain conclusions."

Conclusion: We have revised the Conclusion to clarify that the framework "incorporates applicable evidence from the conventional lithotomy position and general surgical positioning where direct evidence was lacking," and we have updated future research directions to prioritize generating direct evidence for the modified lithotomy position.

We believe these revisions transparently address the reviewer's concern, clearly distinguish between direct and indirect evidence, and explain the applicability rationale without artificially imposing a formal evidence hierarchy that was not part of the original methodological design.

Reviewer Comment 2: The study selection process should be presented clearly in a PRISMA flow diagram. According to the manuscript, 716 records were initially identified, 391 remained after duplicate removal, 373 were excluded after title and abstract screening, leaving 17 publications ultimately included. The reason for exclusion of the remaining record should be specified. More generally, the reasons for exclusion at each stage, particularly after fulltext assessment, should be clearly reported. The authors should also explain why several general surgical positioning references were included despite the stated inclusion criterion of modified lithotomy positioning during laparoscopic colorectal surgery.

Response: We thank the reviewer for these important methodological comments. We have addressed each aspect of this comment as follows:

2.1. PRISMA flow diagram: We have added a PRISMA 2020 flow diagram as Figure 1 to clearly present the study selection process at each stage, following the standard visual format. The diagram depicts the complete flow from identification (N=716) through screening, eligibility assessment, and final inclusion (N=17), including all exclusion points.

2.2. Missing data point and fulltext exclusion clarified: We apologize for the omission in the original manuscript. The reviewer correctly identified that the data chain was incomplete. The correct numbers are: of the 391 records remaining after deduplication, 373 were excluded at the title and abstract stage, leaving 18 records that underwent fulltext assessment. One record was excluded at the fulltext stage because it did not clearly describe surgical positioning, making it impossible to determine its applicability to the modified lithotomy position or related positioning contexts. Consequently, 17 publications were ultimately included. We have revised the Methods section to explicitly report these corrected numbers.

2.3. Table 1 — Fulltext exclusion documented: We have added Table 1 documenting the record excluded at the fulltext assessment stage, including the author, year, and specific reason for exclusion ("Did not clearly describe surgical positioning; the positioning method was inadequately reported to determine applicability to the modified lithotomy position or related surgical positioning contexts"). This addresses the reviewer's request that the reason for exclusion of the remaining record be specified.

2.4. Reasons for exclusion at each stage clearly reported: We have revised the Methods section to clearly report the reasons for exclusion at each stage:

Identification stage: 325 duplicate records were removed.

Screening stage: 373 records were excluded after title and abstract screening due to wrong population (e.g., pediatric or noncolorectal surgery), wrong intervention (e.g., nonlithotomy positioning without transferable mechanisms), or wrong study design (e.g., narrative reviews or case reports not meeting the PIPOST criteria).

Eligibility stage: 1 record was excluded after fulltext assessment because it did not clearly describe surgical positioning.

2.5. Inclusion of general surgical positioning references: Please see our detailed response to Comment 1 above. In brief, we have revised the PIPOST intervention criteria, Introduction, and Discussion to explicitly explain that applicable evidence from the conventional lithotomy position and general surgical positioning was included where anatomical, physiological, or pathophysiological mechanisms were transferable to the modified lithotomy context. This pragmatic extension of the evidence base was necessitated by the limited volume of direct evidence specific to the modified lithotomy position in laparoscopic colorectal surgery. The PRISMA flow diagram also includes a note clarifying the evidence scope of the included studies.

Reviewer Comment 3: The registration status of this review should be clarified. The manuscript states that the study was registered with the Fudan University EvidenceBased Nursing Center (registration number: ER20259536), but it is unclear whether this registration was prospective or retrospective. If the registration was completed after the commencement of the review, this should be explicitly disclosed, along with an explanation of the circumstances and confirmation that no substantive methodological amendments were made between the start of screening and the registration date.

Response: We thank the reviewer for raising this important issue regarding research transparency. We acknowledge that the registration was indeed retrospective, and we apologize for not having made this explicit in the original manuscript. We have revised the manuscript to provide full transparency regarding the registration status as follows:

Methods — Protocol and Registration: We have revised this subsection to state: "The review protocol was registered with the Fudan University EvidenceBased Nursing Center (registration number: ER20259536) on December 15, 2025.

Abstract: We have added the following statement to the Abstract: "Registration: This review was registered with the Fudan University EvidenceBased Nursing Center (registration number ER20259536; registration date: December 15, 2025. This registration was retrospective."

We confirm that the review protocol was fully finalized before the commencement of literature screening, and no substantive changes to the methodology (including the research question, PIPOST framework, search strategy, inclusion/exclusion criteria, or quality appraisal methods) were made between the start of screening and the registration date. The retrospective registration does not affect the methodological rigor or the validity of the findings presented in this review. We appreciate the reviewer's emphasis on transparency, and we believe these revisions appropriately address this concern.

Reviewer Comment 4: Although the total number of included publications is reported as 17, the reference numbers and composition of the included studies are not consistent among the Results section, Table 1, and Table 2. For example, the guideline references listed in the Results do not fully correspond to those presented in the tables. In addition, the Abstract reports 20 evidence points, whereas Table 5 and the Discussion describe 18 recommendations. The authors should carefully recheck the entire process of study selection, quality assessment, evidence extraction, and evidence synthesis, and ensure consistency among the main text, tables, and reference numbering.

Response: We thank the reviewer for this careful observation. We have thoroughly rechecked the entire process of study selection, quality assessment, evidence extraction, and evidence synthesis. All inconsistencies among the Results section, tables, and reference numbering have been carefully corrected and verified. Specifically, the reference numbers and composition of included studies are now fully consistent across the Results, Table 1, and Table 2; and the number of recommendations has been unified to 18 throughout the Abstract, Table 5, and the Discussion. We apologize for these oversights and confirm that the final manuscript is internally consistent.

We hope that these revisions satisfactorily address all of the reviewers' concerns and strengthen the methodological transparency and rigor of our manuscript. We appreciate the opportunity to improve the quality of our work.

Sincerely,

Jiye SANG

1057067874@qq.com

Attachment Submitted filename: renamed_d07d9.docx

# Peer review: Decision Letter 1

**DOI:** 10.1371/journal.pone.0360728.r003

**Authors:** Tsutomu Kumamoto

14 Sep 2026

Dear Dr. SANG,

Thank you for submitting your manuscript to PLOS One. After careful consideration, we feel that it has merit but does not fully meet PLOS One’s publication criteria as it currently stands. Therefore, we invite you to submit a revised version of the manuscript that addresses the points raised during the review process.

Please submit your revised manuscript by Oct 29 2026 11:59PM. If you will need more time than this to complete your revisions, please reply to this message or contact the journal office at plosone@plos.org. When you're ready to submit your revision, log on to https://www.editorialmanager.com/pone/ and select the 'Submissions Needing Revision' folder to locate your manuscript file.

- A letter that responds to each point raised by the academic editor and reviewer(s). You should upload this letter as a separate file labeled 'Response to Reviewers'.
- A marked-up copy of your manuscript that highlights changes made to the original version. You should upload this as a separate file labeled 'Revised Manuscript with Track Changes'.
- An unmarked version of your revised paper without tracked changes. You should upload this as a separate file labeled 'Manuscript'.

If you would like to make changes to your financial disclosure, please include your updated statement in your cover letter. Guidelines for resubmitting your figure files are available below the reviewer comments at the end of this letter.

If applicable, we recommend that you deposit your laboratory protocols in protocols.io to enhance the reproducibility of your results. Protocols.io assigns your protocol its own identifier (DOI) so that it can be cited independently in the future. For instructions see: https://journals.plos.org/plosone/s/submission-guidelines#loc-laboratory-protocols. Additionally, PLOS One offers an option for publishing peer-reviewed Lab Protocol articles, which describe protocols hosted on protocols.io. Read more information on sharing protocols at https://plos.org/protocols?utm_medium=editorial-email&utm_source=authorletters&utm_campaign=protocols.

As the corresponding author, your ORCID iD is verified in the submission system and will appear in the published article. PLOS supports the use of ORCID, and we encourage all coauthors to register for an ORCID iD and use it as well. Please encourage your coauthors to verify their ORCID iD within the submission system before final acceptance, as unverified ORCID iDs will not appear in the published article. *Only*  the individual author can complete the verification step; PLOS staff *cannot*  verify ORCID iDs on behalf of authors.

We look forward to receiving your revised manuscript.

Kind regards,

Tsutomu Kumamoto

Academic Editor

PLOS One

Journal Requirements:

1. If the reviewer comments include a recommendation to cite specific previously published works, please review and evaluate these publications to determine whether they are relevant and should be cited. There is no requirement to cite these works unless the editor has indicated otherwise.

2. Please review your reference list to ensure that it is complete and correct. If you have cited papers that have been retracted, please include the rationale for doing so in the manuscript text, or remove these references and replace them with relevant current references. Any changes to the reference list should be mentioned in the rebuttal letter that accompanies your revised manuscript. If you need to cite a retracted article, indicate the article’s retracted status in the References list and also include a citation and full reference for the retraction notice.

**Additional Editor Comments:**

The manuscript has been substantially and appropriately revised, and the previous concerns have been largely addressed.

Only one point requires clarification regarding the response to Reviewer Comment 3. In the response letter, the authors state that the registration was retrospective, whereas the Methods section describes the protocol as prospectively registered prior to literature screening and data extraction. Please confirm the actual registration timing and revise the relevant statements accordingly to ensure consistency between the response letter and the manuscript.

Other than this point, the manuscript has been satisfactorily revised.

Reviewers' comments:

Reviewer's Responses to Questions

**Comments to the Author**

Reviewer #1: All comments have been addressed

**********

2. Is the manuscript technically sound, and do the data support the conclusions?

Reviewer #1: Yes

**********

3. Has the statistical analysis been performed appropriately and rigorously?

Reviewer #1: Yes

**********

4. Have the authors made all data underlying the findings in their manuscript fully available?

The PLOS Data policy

Reviewer #1: Yes

**********

5. Is the manuscript presented in an intelligible fashion and written in standard English?

Reviewer #1: Yes

**********

Reviewer #1: The authors have done a commendable job in addressing all the reviewers' comments. The manuscript’s methodology and structure have been substantially strengthened.Before final publication,

please address the following minor cosmetic and proofreading

corrections:References 9–13: Please update the proxy/institutional URLs (e.g., uptodate.tsyxy...) to standard, official UpToDate URLs or standard citation formats.

Reference 27: The citation details at the end are incomplete. Please provide the full citation (all author names, article title, journal, and year).

Figure 1 (PRISMA Flowchart):Correct the header typo from "Identifacation" to "Identification". Fix the duplicated label in the middle flow boxes ("Records after duplicate removal").

Overall, excellent work, and the manuscript is ready for publication subject to these minor fixes

**********

what does this mean?). If published, this will include your full peer review and any attached files.

If you choose “no”, your identity will remain anonymous but your review may still be made public.

**Do you want your identity to be public for this peer review?**  For information about this choice, including consent withdrawal, please see our Privacy Policy

Reviewer #1: **Yes:**  Rozana Hossam Eldeen zayed

To ensure your figures meet our technical requirements, please review our figure guidelines: https://journals.plos.org/plosone/s/figures

You may also use PLOS’s free figure tool, NAAS, to help you prepare publication quality figures: https://journals.plos.org/plosone/s/figures#loc-tools-for-figure-preparation.

NAAS will assess whether your figures meet our technical requirements by comparing each figure against our figure specifications.

# Author comment: Author response to Decision Letter 2

**DOI:** 10.1371/journal.pone.0360728.r004

15 Sep 2026

Response to Reviewer #1:

We thank Reviewer #1 for the positive evaluation and for the helpful minor corrections.

Comment 1: References 9–13: Please update the proxy/institutional URLs to standard, official UpToDate URLs or standard citation formats.

Response: All proxy/institutional URLs have been removed and replaced with official UpToDate URLs, formatted in PLOS ONE (Vancouver) style:

9. Welch MB. Patient positioning for surgery and anesthesia in adults [Internet]. UpToDate; 2024 Nov 5 [cited 2026 Sep 14]. Available from: https://www.uptodate.com/contents/patient-positioning-for-surgery-and-anesthesia-in-adults

10. Hemmer LB. Neuromonitoring in surgery and anesthesia [Internet]. UpToDate; [cited 2026 Sep 14]. Available from: https://www.uptodate.com/contents/neuromonitoring-in-surgery-and-anesthesia

11. Hammerberg EM. Acute compartment syndrome of the extremities [Internet]. UpToDate; 2025 Aug 12 [cited 2026 Sep 14]. Available from: https://www.uptodate.com/contents/acute-compartment-syndrome-of-the-extremities

12. Chung KC. Pathophysiology, classification, and causes of acute extremity compartment syndrome [Internet]. UpToDate; 2023 Dec 13 [cited 2026 Sep 14]. Available from: https://www.uptodate.com/contents/pathophysiology-classification-and-causes-of-acute-extremity-compartment-syndrome

13. Newman NJ, Roth S. Postoperative visual loss after anesthesia for nonocular surgery [Internet]. UpToDate; [cited 2026 Sep 14]. Available from: https://www.uptodate.com/contents/postoperative-visual-loss-after-anesthesia-for-nonocular-surgery

Comment 2: Reference 27: The citation details at the end are incomplete.

Response: Reference 27 has been completed as follows:

27. Shi R, Zhang R, Li HY, He JQ, Chen YC, An JJ, et al.; Guangdong Nursing Association Operating Room Nursing Committee. Expert consensus on whole-process management of intraoperatively acquired pressure injuries in the operating room. Journal of Nursing. 2024;31(19):58–64. DOI: 10.16460/j.issn1008-9969.2024.19.058.

Comment3: Figure 1 (PRISMA Flowchart): “Identifacation” → “Identification”.

Response: The typo has been corrected to “Identification” in the revised Figure 1.

Comment 4: Fix the duplicated label “Records after duplicate removal” in the middle flow boxes.

Response: The duplicated label has been removed; Figure 1 now contains a single correct “Records after duplicate removal” box and accurately represents the screening process. The revised figure has been uploaded.

All authors have reviewed and approved the revised manuscript. We confirm there are no conflicts of interest. Thank you again for your time and guidance

Attachment Submitted filename: renamed_2282a.docx

# Editor report: Decision Letter 2

**DOI:** 10.1371/journal.pone.0360728.r005

**Authors:** Tsutomu Kumamoto

30 Sep 2026

Systematic Review and Best Evidence Summary of Patient Positioning Management in the Modified Lithotomy Position during Laparoscopic Colorectal Surgery

PONE-D-26-20549R2

Dear Dr. Jiye SANG,

We’re pleased to inform you that your manuscript has been judged scientifically suitable for publication and will be formally accepted for publication once it meets all outstanding technical requirements.

Within one week, you’ll receive an e-mail detailing the required amendments. When these have been addressed, you’ll receive a formal acceptance letter and your manuscript will be scheduled for publication.

An invoice will be generated when your article is formally accepted. Please note, if your institution has a publishing partnership with PLOS and your article meets the relevant criteria, all or part of your publication costs will be covered. Please make sure your user information is up-to-date by logging into Editorial Manager at Editorial Manager® and clicking the ‘Update My Information' link at the top of the page. For questions related to billing, please contact billing support.

If your institution or institutions have a press office, please notify them about your upcoming paper to help maximize its impact. If they’ll be preparing press materials, please inform our press team as soon as possible -- no later than 48 hours after receiving the formal acceptance. Your manuscript will remain under strict press embargo until 2 pm Eastern Time on the date of publication. For more information, please contact onepress@plos.org.

Kind regards,

Tsutomu Kumamoto

Academic Editor

PLOS One

# Editor report: Acceptance letter

**DOI:** 10.1371/journal.pone.0360728.r006

**Authors:** Tsutomu Kumamoto

PONE-D-26-20549R2

PLOS One

Dear Dr. SANG,

I'm pleased to inform you that your manuscript has been deemed suitable for publication in PLOS One. Congratulations! Your manuscript is now being handed over to our production team.

At this stage, our production department will prepare your paper for publication. This includes ensuring the following:

* All references, tables, and figures are properly cited

* All relevant supporting information is included in the manuscript submission,

* There are no issues that prevent the paper from being properly typeset

You will receive further instructions from the production team, including instructions on how to review your proof when it is ready. Please keep in mind that we are working through a large volume of accepted articles, so please give us a few days to review your paper and let you know the next and final steps.

Lastly, if your institution or institutions have a press office, please let them know about your upcoming paper now to help maximize its impact. If they'll be preparing press materials, please inform our press team within the next 48 hours. Your manuscript will remain under strict press embargo until 2 pm Eastern Time on the date of publication. For more information, please contact onepress@plos.org.

You will receive an invoice from PLOS for your publication fee after your manuscript has reached the completed accept phase. If you receive an email requesting payment before acceptance or for any other service, this may be a phishing scheme. Learn how to identify phishing emails and protect your accounts at https://explore.plos.org/phishing.

If we can help with anything else, please email us at customercare@plos.org.

Thank you for submitting your work to PLOS One and supporting open access.

Kind regards,

PLOS One Editorial Office Staff

on behalf of

M.D., Ph.D. Tsutomu Kumamoto

Academic Editor

PLOS One
